# *O*-GlcNAc Modification During Pregnancy: Focus on Placental Environment

**DOI:** 10.3389/fphys.2018.01263

**Published:** 2018-09-12

**Authors:** Victor Vitorino Lima, Vanessa Dela Justina, Rinaldo Rodrigues dos Passos, Gustavo Tadeu Volpato, Paula Cristina S. Souto, Sebastian San Martin, Fernanda Regina Giachini

**Affiliations:** ^1^Institute of Health and Biological Science, Federal University of Mato Grosso, Barra do Garças, Brazil; ^2^Institute of Biological Science, Federal University of Goias, Goiânia, Brazil; ^3^Biomedical Research Center, School of Medicine, Universidad de Valparaíso, Valparaíso, Chile

**Keywords:** placental dysfunction, *O*-GlcNAc, post-translational modification, pregnancy, placentation

## Abstract

Successful placentation is a key event for fetal development, which commences following embryo implantation into the uterine wall, eliciting decidualization, placentation, and remodeling of blood vessels to provide physiological exchange between embryo-fetus and mother. Several signaling pathways are recruited to modulate such important processes and specific proteins that regulate placental function are a target for the glycosylation with *O-*linked β-N-acetylglucosamine (*O-*GlcNAc), or *O-*GlcNAcylation. This is a reversible post-translational modification on nuclear and cytoplasmic proteins, mainly controlled by *O-*GlcNAc transferase (OGT) and *O-*GlcNAcase (OGA). *O-*GlcNAcylation has been implicated as a modulator of proteins, both in physiological and pathological conditions and, more recently, *O*-GlcNAc has also been shown to be an important modulator in placental tissue. In this mini-review, the interplay between *O-*GlcNAcylation of proteins and placental function will be addressed, discussing the possible implications of this post-translational modification through placental development and pregnancy.

## Introduction

The hemochorial placenta allows blood coming from the maternal circulation to directly contact the fetal chorion, favoring nutrient exchange to the embryo and fetus (Croy et al., 2009). In addition to providing a complete environment for embryo-fetal development, this non-innervated organ also has important implications in endocrine and physiological control during pregnancy. These features display a tightly regulated placentation process requiring precise mechanisms to modulate embryo implantation, decidualization of the endometrium and uterine blood vessel remodeling to generate a functional placenta (Maltepe et al., 2010).

The placenta displays crucial functions during pregnancy, and its performance is associated with morphological integrity, providing the desirable environment for fetal development. Several maternal factors may impact placental function, including co-existence of diabetes, hypertension, and other conditions. Metabolic homeostasis, together with the development of gestational immune tolerance (Elliot and Crespi, 2006; Croy et al., 2009), favor an environment without stressors, and are requirements for successful placental development.

*O-*GlcNAcylation is a reversible and dynamic post-translational modification with *O-*linked β-N-acetylglucosamine (*O-*GlcNAc), targeting cytoplasmic and nuclear proteins at serine, threonine and tyrosine (Ser-Thr-Tyr) residues. This process occurs in several proteins in eukaryotic cells, and is analogous to protein phosphorylation (Lima et al., 2012; van der Laarse et al., 2018). Unlike other post-translational modifications, *O*-GlcNAc is regulated exclusively by two enzymes: *O-*GlcNAc transferase (OGT), which catalyzes the β -attachment of *O-*GlcNAc to the hydroxy groups of Ser-Thr-Tyr residues; and β-N-acetylglucosaminidase (OGA, or *O-*GlcNAcase), which catalyzes the hydrolytic cleavage of *O-*GlcNAc from proteins (Laczy et al., 2009; Lima et al., 2011). Interestingly, OGA and OGT are extensively expressed in placentas (Lubas et al., 1997; Gao et al., 2001) and several proteins that play important roles in placental function are targets for *O-*GlcNAcylation.

**FIGURE 1:**
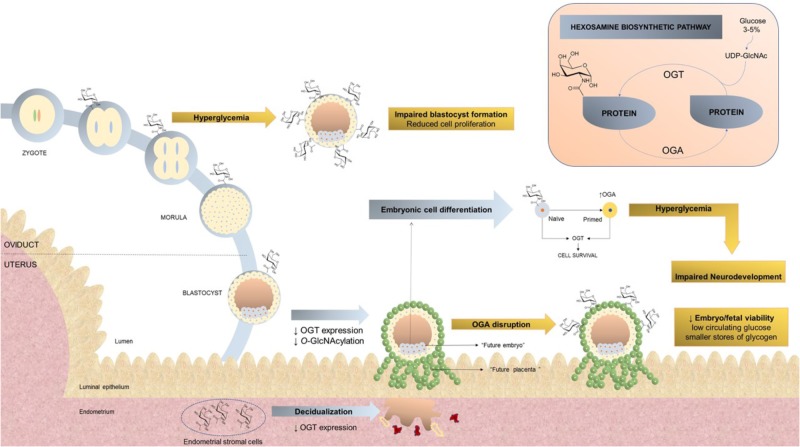
Hexosamine biosynthetic pathway and *O*-glycosylation with *O-*linked β-N-acetylglucosamine during pregnancy. (Pink rectangle) Around 3–5% of intracellular glucose is converted through the HBP to substrate for enzymes involved in the synthesis of UDP-GlcNAc, consequently leading to protein modification through *O*-GlcNAc. OGT catalyzes the addition of *O-*GlcNAc to proteins, whereas OGA removes *O-*GlcNAc from proteins. (Bigger figure) Under physiological conditions, *O*-GlcNAc modifications to intracellular proteins are essential for embryogenesis, promoting the function and viability of various cell types. This process may be disrupted under specific conditions, including hyperglycemia or OGA disruption, where an increase in *O*-GlcNAc is observed, with a consequent reduction in cell proliferation, impaired blastocyst formation and decreased embryo viability. Abbreviations: HBP, Hexosamine biosynthetic pathway; *O*-GlcNAc, glycosylation with *O-*linked β-N-acetylglucosamine; OGA, β-N-acetylglucosaminidase; OGT, *O-*GlcNAc transferase.

The most important source for *O-*GlcNAc formation is the hexosamine biosynthetic pathway (HBP). After glucose uptake, glucose can either be used in the glycolytic pathway or the HBP. The HBP uses fructose 6-phosphate to form glucosamine 6-phosphate, with glutamine serving as the donor of the amino group, whereas this reaction is catalyzed via glutamine: fructose 6-phosphate aminotransferase (GFAT), which is rapidly acetylated through the action of acetyl-CoA:d-glucosamine-6-phosphate N-acetyltransferase (GAT) and isomerized to *N*-acetylglucosamine-1-phosphate (GlcNAc-1-P). UDP-GlcNAc pyrophosphorylase then converts GlcNAc-1-P to UDP-GlcNAc, which serves as the donor for *O*-GlcNAc when OGT is activated. Glucosamine can also enter the cell through glucose transporters and is rapidly phosphorylated by hexokinase yielding glucosamine 6-phosphate, thereby passing the rate-limiting first step of the HBP (**Figure 1**; Lima et al., 2012).

In this review, we will present evidence of how this post-translational modification interacts with proteins that are important for placental function, discussing possible implications in pregnancy.

## *O-*GlcNAc During Pregnancy

### *O*-GlcNAc Role During Pre-implantation, Implantation, and Embryo Development

Several stages of pregnancy may be affected by *O-*GlcNAc (**Figure 1**). Fast trophoblast proliferation, along with the development of the chorionic sac and chorionic villi marks the earliest stage of placental formation (Rossant and Cross, 2001). Maternal hyperglycemia may negatively impact pre-implantation by reducing the embryo’s ability for glucose uptake, favoring miscarriage and congenital anomalies (Jungheim and Moley, 2008; Damasceno et al., 2017). In hyperglycemia and glucosamine incubation increased *O-*GlcNAcylation occurs, resulting in reduced cell proliferation and, therefore, impaired blastocyst formation, as demonstrated in an experimental model of mouse zygote cultures. Inhibition of OGT was able to partially restore cell proliferation and blastocyst formation under hyperglycemic conditions, suggesting that dysregulation of both HBP and *O-*GlcNAcylation may favor embryotoxic effects during pre-implantation development (Pantaleon et al., 2010). *O-*GlcNAc displays an important role not only in trophoblast proliferation, but also in embryonic cell differentiation. Naïve mouse embryonic stem cells, derived from pre-implantation embryos, maintain an undifferentiated state via augmented *O-*GlcNAc expression (Shi et al., 2013). The differentiation process from naïve to a primed state, as observed in cells from post-implantation embryos, requires OGA expression, whereas OGT expression is observed in maintenance of cell survival (Jang et al., 2012; Shi et al., 2013; Speakman et al., 2014). Interestingly, OGT also contributes to cell survival of primed embryo cells (O’Donnell et al., 2004) and expression of both OGT and OGA is required to revert primed to naïve cells (Miura and Nishihara, 2016). Later, it was demonstrated that the histone variant H2A is a target for *O-*GlcNAc at Ser^40^, and this post-translational modification is required for H2A to contribute to the trophoblast stem cell differentiation process, being correlated with the evolution of placental tissue (Hirosawa et al., 2016). Indeed, H2A is required during mouse embryonic stem cell differentiation, allowing these cells to change gene expression. H2A is also highly expressed in early placental development (Kafer et al., 2015).

Post-translational modifications of histones represent an important mechanism of DNA damage repair. An example is the phosphorylation of the histone H2AX, of itself an effective repair mechanism. This process is, however, restrained to small compartments, and must occur within a limited time frame. *O*-GlcNAcylation also occurs in H2AX, limiting the expansion of DNA damage-induced phosphorylation of chromatin (Chen and Yu, 2016). Embryos from diabetic mothers display exacerbated DNA damage, evidenced by the co*-*localization of H2AX. *O-*GlcNAcylation was observed in diabetic blastocyst-stage embryos, favoring impairment of pre-implantation embryo development (Brown et al., 2018). That precursors coming from the metabolic flux have the ability to modulate nuclear function has been denominated metaboloepigenetics (Donohoe and Bultman, 2012); this mechanism provides evidence for an epigenetic contribution to the vertical transmission of diabetes.

OGA expression is also related to embryo/fetal viability since *Oga* gene disruption results in augmented levels of global *O-*GlcNAcylation. These genetically modified animals display high perinatal mortality, associated with low circulating glucose levels and smaller stores of glycogen in the liver. In this experimental model, other metabolic alterations were identified in heterozygous mice, including fat accumulation, reduced insulin sensitivity, glucose intolerance and hyperleptinemia. *Oga* disruption generated defective metabolic homeostasis, contributing to obesity and insulin resistance (Keembiyehetty et al., 2015).

Successful placentation involves decidual cells that encapsulate the implanting embryo, providing nutrition and favoring an immunological environment that allows trophoblast invasion and, later, placentation. It is mandatory for decidual cells to develop mechanisms to block stressor signals, favoring the integrity of this initial stage of the fetal-maternal interface (Weimar et al., 2012; Erlebacher, 2013). Decidualization of primary endometrial stromal cells results in reduced global *O-*GlcNAcylation, mediated by decreased OGT expression, without changes in OGA expression. Cell differentiation occurs simultaneously with enhanced expression of epidermal growth factor domain–specific *O-*linked GlcNAc transferase (EOGT), involving mechanisms of energy homeostasis and glucose and fatty acid metabolism, which explains, at least in part, adverse pregnancy outcomes observed in metabolic disorders (Muter et al., 2018).

*O*-GlcNAcylation impacts embryogenesis and alterations in the maternal glucose metabolism may disrupt neurodevelopment. Neural stem cells submitted to an *in vitro* hyperglycemic environment resulted in augmented global *O-*GlcNAcylation via OGT enhanced activity, and displayed neural tube defects, suggesting that inhibition of altered OGT might be beneficial in preventing birth defects in hyperglycemic pregnancies (Kim et al., 2017). Given the importance of this pathway on neurodevelopment, human embryonic stem cells were used to demonstrate that augmented global *O-*GlcNAcylation is associated with reduced neural progenitor proliferation and premature differentiation of cortical neurons. Therefore, *O-*GlcNAc regulation may represent an important mechanism observed in metabolically compromised pregnancies, contributing to neuronal impairment in the offspring (Parween et al., 2017).

### *O*-GlcNAc and Immune System During Pregnancy

The embryo is recognized as non-self by the maternal immune system, and therefore, several adaptations are required to prevent rejection during implantation (Robertson and Moldenhauer, 2014). One important role of natural killer (NK) cells is to destroy cells that fail to express major histocompatibility complex (MHC) class I molecules; during pregnancy, in contrast to their primary function, placental NK cells tolerate cells from fetal tissue, which do not express maternal MHC I molecules (Ljunggren and Karre, 1990; King and Loke, 1991). A possible explanation for the tissue-specific behavior of placental NK cells is that non-classical MHC I molecules, including human leukocyte antigen-G (HLA-G), are expressed in fetal extra villous trophoblasts and may inhibit NK cell cytotoxicity during pregnancy (Carosella et al., 1999; Kumpel and Manoussaka, 2012). Trophoblast cells present in the maternofetal interface secrete a soluble HLA-G1 (sHLA-G1) isoform in the amniotic fluid, and are released into the maternal circulation, favoring systemic immunoinhibitory activity (McMaster et al., 1998). Indeed, sHLA-G1 secreted by syncytiotrophoblast specifically induces the apoptosis of CD8+ T cells (Solier et al., 2002). Interestingly, NK cell cytotoxicity occurs simultaneously with reduced *O-*GlcNAcylation and seems to be inhibited by the sHLA-G1 α chain via an *O-*GlcNAc dependent-mechanism. When NK92 cells, an NK cell line, were submitted to a cytotoxicity assay using K562 cells as target cells, *O-*GlcNAc levels decreased inversely to cytotoxic activity (Yao et al., 2004). Preincubation with GST-HLA-Glα chain prevented the decrease of *O-*GlcNAc levels, reverting NK92 cytotoxicity.

Cytokine production is also modulated by *O*-GlcNAcylation. Augmented *O-*GlcNAc levels in the placenta during hyperglycemia coincided with augmented placental levels of interleukin (IL)-6 and tumor necrosis factor alpha (TNF-α). Interestingly, both cytokines positively correlated with placental weight, and negatively correlated with fetal weight and placental efficiency in hyperglycemic conditions (Dela Justina et al., 2017). Reduced embryo implantation and impaired blastocyst development was observed in an experimental mouse model of diabetes. These findings were related to an inflammatory imbalance, elicited by increased expression of pro-inflammatory cytokines in the uterus, including IL-1α, TNF-α, and interferon gamma (IFNγ), and a pronounced reduction of anti-inflammatory regulatory T-cells within the uterus-draining lymph nodes (Brown et al., 2018).

### *O*-GlcNAC Regulation of Transcriptional Factors in Placental Tissue

Post-translational modifications are important key regulators of transcription factors. Considering the high occurrence of *O-*GlcNAcylation of nuclear proteins, *O-*GlcNAc has been implicated to be a major modulator of transcriptional activity (Ozcan et al., 2010). The first transcription factor described to be a target for *O-*GlcNAc modulation was specific protein 1 (Sp-1), a member of the Sp factors; removal of *O-*GlcNAc favored protein association, as demonstrated in HeLa cells (Roos et al., 1997).

Later, it was demonstrated that transcription factors from the placenta are also targets of *O-*GlcNAc modulation. The oncofetal protein gene (Pem) is expressed in a stage-specific manner during murine embryogenesis in placental, but not adult, tissues (Wilkinson et al., 1990). Murine placenta and embryonic expression of Pem is highly regulated, involving E74 like Ets transcription factor 1 (Elf-1) and Sp-1 transcription factors (Rao et al., 2002). Efl-1, which belongs to the Ets family, and Sp-1 are, similarly, modulated by *O-*GlcNAc (Jackson and Tjian, 1988; Juang et al., 2002; Chu and Ferro, 2005). Sp-1 and Elf-1 activate Pem promoter elements, favoring its transcription (Rao et al., 2002). *In vitro*, DNA binding and competition assays have demonstrated that *O-*GlcNAcylation of Sp-1 does not affect DNA binding itself, but reduces the ability of Sp-1 to interact with Elf-1. As a result, the activation of the Pem promoter element is reduced, and, thus, Pem expression (Lim and Chang, 2009). This was the first work to provide evidence that *O-*GlcNAc modulates transcription factors that regulate genes specifically expressed during embryogenesis, displaying a placental distribution.

The hypoxia-inducible factor-1alpha (HIF-1α), is a subunit of a heterodimeric transcription factor, hypoxia-inducible factor 1 (HIF-1), which is stabilized at the protein level in response to hypoxia. This transcription factor plays an essential role during vascular development of the placenta and OGA favors HIF-1α stabilization. OGA deficient mice display elevated *O-*GlcNAcylation along with defective placental vasculogenesis, characterized by reduced vasculature in the labyrinth region, directly contributing to fetal growth restriction. Interestingly, in this model OGA deletion reduced OGT activity, which resulted in HIF-1α suppression, reducing transcriptional activation of target genes (Yang et al., 2015). This evidences an important role of *O-*GlcNAcylation in cellular stress conditions. During proliferation, OGT deletion, which reduces *O-*GlcNAc levels, modulates HIF-1α favoring ER stress-mediated apoptosis, as observed in cancer cells (Ferrer et al., 2014).

Under hyperglycemic conditions, *O-*GlcNAc modifications also occur in nuclear factor kappa-light-chain-enhancer of activated B cells (NF-κB), found in rat placental tissue (Dela Justina et al., 2017). This represents non-canonical activation of NF-κB, also described in other cells (Yang et al., 2008), in which augmented levels of *O-*GlcNAc bind to NF-κB, favoring its translocation to the nucleus, augmenting production of pro*-*inflammatory cytokines (Dela Justina et al., 2017).

### *O*-GlcNAc and Maternal Stress

**FIGURE 2:**
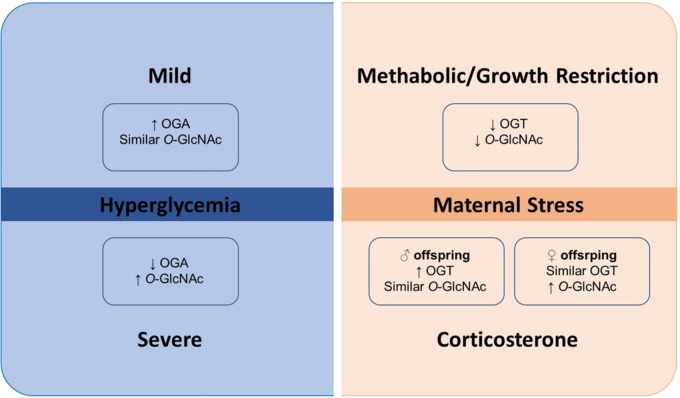
Metabolic maternal stress and *O*-GlcNAc. *O*-GlcNAcylation appears to act as a stress sensor since it exerts its fundamental effects in response to stress. OGT has also been identified as a placental biomarker of cellular stress. During metabolic maternal stress and growth restriction, both OGT and *O-*GlcNAcylation were significantly lower. Placentas of female mice offspring had higher basal OGT expression compared to placentas of male offspring. However, following exposure to corticosterone, OGT expression raised in male placentas and remained the same in female placentas, simultaneously with increased global *O-*GlcNAcylation in female placentas, which was unmodified in male placentas. Abbreviations: *O*-GlcNAc, glycosylation with *O*-linked β-N-acetylglucosamine; OGA, β-N-acetylglucosaminidase; OGT, *O*-GlcNAc transferase.

In several cell types, *O-*GlcNAcylation provides an important tool for cell survival in conditions of elevated stressors, and several forms of cellular injury result in dynamic changes to *O-*GlcNAcylation (Martinez et al., 2017). During pregnancy, exposure to stress contributes to maternal and fetal metabolic alterations and reduces placental growth. Both maternal stress and growth restriction have been consistently associated with metabolic dysfunction observed in offspring (**Figure 2**). High glucose levels, observed in mothers submitted to experimental models of stress, impact the gene expression profile of offspring. OGT is one of the genes affected and, in conditions of maternal stress, offspring display reduced OGT expression in the labyrinth region (Briffa et al., 2017).

A genome-wide array approach identified OGT as a cellular stress marker during pregnancy. Both OGT and *O-*GlcNAcylation were significantly lower when mothers were submitted to prenatal stress. In addition to being a biomarker of stress, OGT was also shown to be crucial for neurodevelopment, and its expression was affected to a greater extent in male, compared to female offspring (Howerton et al., 2013). Physiologically, *O-*GlcNAcylation is greatly decreased during early development and the shortest isoform of OGT (sOGT) is essentially undetectable during early development, but increases consistently after birth (Liu et al., 2012). To address the direct impact of placental OGT in programming the developing brain, an elegant study was conducted in a transgenic mouse model with targeted placental disruption of OGT (PI-OGT). Offspring from this model were compared to those of a mouse model of early prenatal stress (Howerton and Bale, 2014). These experiments confirmed that OGT is an important placental biomarker of maternal stress, resulting in a long-term harmful effect on offspring, which is suitable for metabolic and neurodevelopmental programing under these conditions.

It appears that OGT expression may change according to the kind and intensity of stress experienced by the mother, and affect offspring in a sexually dimorphic manner. Physiologically, the placenta of female mice offspring had higher basal OGT expression, compared to placentas of males. Following corticosterone exposure, OGT expression increased in placentas of males, but global *O-*GlcNAcylation was not modified, whereas, in placentas of females, OGT expression remained the same, and increased global *O-*GlcNAcylation was observed. These findings show that placentas from female offspring have a greater capacity to rapidly respond to maternal stress and suggests that offspring are affected by maternal stress in a sexually dimorphic pattern. This may impact future life, where males may be more suitable for diseases that are influenced by intra-uterus environment (Pantaleon et al., 2017). A deleterious consequence of maternal stress on offspring includes depression. Adult female rats displayed sex-specific depressive-like behavior when submitted to an intrauterine stress environment as offspring, while males did not display these symptoms (Liu et al., 2018). Interestingly, when these animals were subjected to a swimming exercise, depression symptoms were ameliorated, and this improvement was associated with OGT-related mitochondrial motility.

Metabolic maternal stress, as observed in placentas during severe hyperglycemia, resulted in increased *O*-GlcNAc levels compared to placentas from control and mildly hyperglycemic rats. OGA expression was reduced in placentas from the severely hyperglycemic rats, whereas augmented OGA was found in placentas from the mild hyperglycemic group, compared to control. No changes in OGT were observed during severe or mild hyperglycemia. *O*-GlcNAc overexpression in hyperglycemic conditions co-exist with placental dysfunction, which was characterized by morphometric alterations along with reduced placental index (Dela Justina et al., 2018).

## Perspectives

The evidence relating *O*-GlcNAc to placental function is at present still limited, mainly due to the limited number of studies conducted so far. OGA and OGT expression and *O*-GlcNAc modification represent important modulator mechanisms involved in placental development. Several pathological conditions result in augmented *O*-GlcNAc levels and may impact these mechanisms, leading to impaired placental development and adversely affect fetal growth. Most of the work conducted has been performed in cell culture or in experimental models of hyperglycemia; future work would be served by evaluating *O-*GlcNAcylation in human placentas. In addition to hyperglycemia, high *O*-GlcNAc levels co-exist in other conditions, including hypertension (Zachara, 2012), kidney injury (Hu et al., 2017), high-fat diet (Lima et al., 2016), obesity (da Costa et al., 2018), cancer (Tiainen et al., 2016), among others. Therefore, a next step would be to verify how the altered *O*-GlcNAc levels observed in various pathological conditions might impact placental development.

The current knowledge on this topic also reveals a potential area for exploration in sexual dimorphism during placentation. This is particularly important at the time of the placental collection in animal studies, with respect to correct sex identification. Hence, it will be exciting to describe whether *O*-GlcNAcylation occurs in a similar pattern at different stages of placentation for male and female offspring.

Several conventional pharmacological and non-pharmacological treatments, including medicinal plants, exercise, among others, are used to improve pregnancy outcomes and fetal growth. It will be interesting to evaluate whether these strategies impact *O*-GlcNAcylation of placental proteins or improve gestational success in pathological conditions.

## Members of the RIVA-TREM

Victor Vitorino Lima, Sebastian San Martin, Paula Cristina S. Souto, Fernanda Regina Giachini (www.rivatrem.org).

## Author Contributions

FG and VL have proposed the topic of this revision and designed the figures. All the authors have contributed to information recruitment, revision design, to write and revise the present version. FG has proposed the topic of this revision and designed the figures. All the authors have contributed to design, write and revise the present version.

## Conflict of Interest Statement

The authors declare that the research was conducted in the absence of any commercial or financial relationships that could be construed as a potential conflict of interest.
